# Draft Genome Sequence of the Fungus *Trametes hirsuta* 072

**DOI:** 10.1128/genomeA.01287-15

**Published:** 2015-11-19

**Authors:** Andrey R. Pavlov, Tatiana V. Tyazhelova, Konstantin V. Moiseenko, Daria V. Vasina, Olga V. Mosunova, Tatiana V. Fedorova, Lilya G. Maloshenok, Elena O. Landesman, Sergei A. Bruskin, Nadezhda V. Psurtseva, Alexei I. Slesarev, Sergei A. Kozyavkin, Olga V. Koroleva

**Affiliations:** a A.N. Bach Institute of Biochemistry, Russian Academy of Sciences, Moscow, Russian Federation; b Fidelity Systems, Inc., Gaithersburg, Maryland, USA; c N.I. Vavilov Institute of General Genetics, Russian Academy of Sciences, Moscow, Russian Federation; d Komarov Botanical Institute, Russian Academy of Sciences, St. Petersburg, Russian Federation

## Abstract

A standard draft genome sequence of the white rot saprotrophic fungus *Trametes hirsuta* 072 (*Basidiomycota*, *Polyporales*) is presented. The genome sequence contains about 33.6 Mb assembled in 141 scaffolds with a G+C content of ~57.6%. The draft genome annotation predicts 14,598 putative protein-coding open reading frames (ORFs).

## GENOME ANNOUNCEMENT

*Trametes hirsuta* strain 072 (LE-BIN 072) (1) is a fungus that has high potential for biotechnological applications, such as industrial production of ligninolytic enzymes for hydrolysis of lignocellulose-containing biomass (2) and for degradation of various xenobiotic compounds and dyes (3). This strain of *T. hirsuta* is typical for Northern Eurasia regions of the Holarctic ecozone, often found in taiga, and usually grows on small fallen trees and branches. The mycelial culture of basidiomycete *T. hirsuta* 072 used in this study was provided by the Collection of the Komarov Botanical Institute (St. Petersburg, Russia). Monokarion isolates (haplonts) were obtained from the dikarion strain of *T. hirsuta* 072 by cultivation for fruit-bodes (4) followed by isolating basidiospores (5). Mating types of 12 single-basidiospore isolates obtained from the fungal basidiomata were characterized by mating the haplonts in all combinations (6), and all four mating types were discovered. The isolate of type A1B1 was then used for sequencing.

Genomic DNA of *Trametes hirsuta* 072 was isolated from the haploid fungal culture after submerged cultivation described earlier (7), using the cetyltrimethylammonium bromide protocol (8), and treated with RNase A.

For Illumina sequencing, a paired-end shotgun library was produced using a TruSeq DNA sample prep kit (Illumina) and three mate-pair libraries (3-kb, 8-kb, and 20-kb spans) were obtained with a Nextera mate pair library sample prep kit (Illumina) and the TruSeq DNA sample prep kit. The shotgun library was then sequenced from both ends on an Illumina HiSeq 2500 (150-cycle run), and the mate-pair libraries were sequenced from both ends on an Illumina MiSeq (250-cycle run).

DNA sequencing resulted in 329.3 million reads from the shotgun library and 35.0 million reads from all mate-pair libraries. The reads were processed for assembly with Newbler 2.6 using scripts written at Fidelity Systems, Inc. For the standard draft assembly containing 33,623,396 bp in 141 scaffolds with 174.5-fold estimated genome coverage, we used 20 million shotgun library reads and 11.5 million reads from the mate-pair libraries. The resulting assembly was screened for contamination by a BLAST (9) search of the NCBI nonredundant database and the UniProt fungal knowledge base.

The GC content in the assembled DNA was found to be about 57.6% as assessed by QUAST (10). Structural and functional gene prediction was produced by Augustus (11) trained on *Laccaria bicolor*. Overall, we found 14,598 putative protein-coding genes, which encompass 798 superfamilies (12); 3,818 genes belong to 2,198 PANTHER families, of which 3,005 genes were annotated to the subfamily level (13). The proteins represented families predominantly involved in transport of substances, carbohydrate metabolism, and metabolic regulation. As predicted by TMHMM 2.0 (14), about 21% of the annotated proteins present putative transmembrane regions, of which 421 have four or more of such regions. As many as 1,013 genes exhibit coiled coils signatures in the translated protein sequences, suggesting involvement of these proteins into protein-protein interactions (15). Also, using SignalP 4.1, we found that 228 translated proteins have a distinct signal peptide sequence and thus are expected to be secreted (16, 17).

### Nucleotide sequence accession numbers.

This whole-genome shotgun project of *Trametes hirsuta* 072 has been deposited at DDBJ/EMBL/GenBank under the accession number LIYB00000000. The version described in this paper is version LIYB01000000.
